# Association between regular walking and periodontitis according to socioeconomic status: a cross-sectional study

**DOI:** 10.1038/s41598-019-49505-2

**Published:** 2019-09-10

**Authors:** Su-Jin Han, Kwang-Hak Bae, Hyo-Jin Lee, Seon-Jip Kim, Hyun-Jae Cho

**Affiliations:** 1 Department of Dental Hygiene, College of Health Science, Gachon University, Incheon, Korea; 2 Oral Health Science Research Centre, Apple tree Dental Hospital, Goyang-si, Gyounggi-do, Korea, Seoul, Korea; 3 Department of Dental Hygiene, College of Dentistry and Research Institute of Oral Science, Gangneung-Wonju National University, Gangeung, Korea; 4 Department of Preventive Dentistry & Public Oral Health, School of Dentistry, Seoul National University, Seoul, Korea; 5 Dental Research Institute, School of Dentistry, Seoul National University, Seoul, Korea

**Keywords:** Dental diseases, Epidemiology

## Abstract

Physical activity reduces the risk and mortality risk of inflammatory diseases. This study aimed to examine the relationship between regular walking and periodontitis in a Korean representative sample of adults according to socioeconomic status. Data acquired by the Sixth Korea National Health and Nutrition Examination Survey in 2014 and 2015 were used. The survey was completed by 11,921 (5,175 males; 6,746 females) participants (≥19 years). Individuals without values on periodontitis were excluded, and 9,728 participants remained. Multivariable logistic regression analysis was done using socio-demographic characteristics (age, gender, income, education), oral health-related variables (flossing, interdental brushing, community periodontal index), oral and general health status and behaviour (smoking, diabetes mellitus), and regular walking. In all models, subjects who walked regularly had significantly lower risks of periodontitis. After adjusting for age, gender, income, education, smoking, diabetes mellitus, flossing, and interdental brushing, the odds ratio for periodontitis in subjects who walked regularly was 0.793 (95% Confidence interval: 0.700–0.898). Non-regular walking groups showed similar social gradients. Risk of low socioeconomic status was not significant in the regular walking group after adjusting for age, gender, income, and education. This study found that regular walking is associated to lower prevalence of periodontitis and can attenuate the relationship between periodontitis and low socioeconomic status.

## Introduction

Periodontitis is an inflammatory chronic disease that leads to the destruction of connective tissue and supporting bone^1,2^. It is a major oral disease that threatens oral health, and its prevalence is increasing mainly because the society is aging^3,4^.

Physical activity provides many health benefits and improves the health-related quality of life^5–7^. Studies have shown that regular physical activity reduces the risk and mortality risk of many systemic diseases including cardiovascular disease, coronary heart disease, colon cancer, diabetes, osteoporosis, obesity, arthritis, and hypertension^8–11^.

Recently, Kortas *et al*.^12^ reported that walking may decrease oxidative stress. Hypertension, obesity, and diabetes mellitus are interlinked in regard to oxidative stress and inflammation^13,14^. Meta-analyses have reported that walking improves the glycaemic control as assessed by glycated haemoglobin (HbA1c) in patients with type 2 diabetes and that aerobic physical activity decreases the blood pressure of subjects with hypertension^15,16^. Oxidative stress can have critical effects on several diseases including periodontitis^17^. Many studies show that regular walking reduces inflammation in the body^18–20^. Therefore, studying the association between regular walking and periodontitis can be valuable.

Some cross-sectional epidemiologic studies confirm the link between physical activity and periodontitis^21,22^. However, there are no systematic large-scale epidemiological studies that have confirmed the effect of walking on periodontal disease. In addition, there is a report showing that the association between periodontitis and other inflammation-related diseases differs according to the socioeconomic status of the patient^23^. Therefore, it is necessary to study socioeconomic status as an effect modifier when studying the association between periodontitis and physical activity.

The objective of this study was to examine the relationship between regular walking and the prevalence of periodontitis in a Korean representative sample of adults according to their socioeconomic status.

## Methods

This study used data acquired in the second and third years (2014–2015) of the Sixth Korea National Health and Nutrition Examination Survey (KNHANES VI). The KNHANES VI was a cross-sectional and nationally representative survey conducted by the Korea Centres for Disease Control and Prevention between 2013 and 2015. Data from the first year (2013) were not used because the variable for regular walking was used only in the second and third years (2014–2015) of the KNHANES VI. Written informed consents were obtained from all subjects with ethical approval by the KCDC Institutional Review Board (IRB number: 2014–12EXP-03-5C, 2015– 01CON-02-6C).

The sampling protocol used was a complex, stratified, multistage probability cluster survey of a representative sample of the non-institutionalized civilian population of Korea. A total of 11,921 participants (5,175 males and 6,746 females), aged 19 years or older, completed the KNHANES VI in 2014 and 2015. Individuals without data on periodontitis and gender were excluded from the analysis. This reduced the sample to 9,728 which was the final number of individuals analysed in this study. From all the data collected by the KNHANES VI, we used the data on socio-demographic characteristics (age, gender, individual income, and level of education), oral health-related variables (dental flossing, interdental brushing, and community periodontal index [CPI]), oral and general health status and behaviour (smoking status and diabetes mellitus), and regular walking.

### Periodontal examination

The periodontal status was evaluated using the CPI developed by the World Health Organization (WHO)^24^. A CPI probe that met the 1997 WHO guidelines was used on ten index teeth, two molars in each posterior sextant, and the upper right and lower left central incisors at six sites per tooth (mesiobuccal, midbuccal, distobuccal, mesiolingual, midlingual, and distolingual), and the periodontal pocket depth was measured. Probing was conducted by dentists who were trained in calibration. Five CPI scores could be recorded: CPI 0, normal; CPI 1, gingival bleeding; CPI 2, presence of gingival calculus; CPI 3, shallow periodontal pocket (>3.5 mm and ≤5.5 mm); and CPI 4, deep periodontal pocket (>5.5 mm). Periodontitis was defined as a CPI score of 3 or 4. Participants were classified into two groups: non-periodontitis and periodontitis.

### Regular walking

The level of physical activity was measured based on the validated Korean version of the International Physical Activity Questionnaire (IPAQ)^25,26^. The specific questions for physical activity were ‘How many days did you walk during the last week?” and “How many minutes did you walk on such a day?’. The respondents were classified as those who regularly walked if they had walked for >30 minutes, ≥5 times a week during the last seven days. The classification followed the KNHANES criteria^27^.

### Covariates

The confounders of this study were the following major socio-demographic factors: gender, age, income, and education. The individual income was classified into four different groups: <25% (the lowest quartile group), 25–49%, 50–74%, and 75–100% (the highest quartile group). The level of education was also classified into four groups based on the Korean education system: below primary school, middle school, high school, and college or higher education. The health behaviour covariates included were smoking, diabetes mellitus, the use of dental floss, and the use of interdental brush. Participants were categorized into two groups based on their smoking experience: ‘never smoker’ and ‘current or past smoker’. With respect to diabetes mellitus, participants were classified into three groups: normal, impaired fasting glucose, and diabetes.

### Statistical analysis

Data were analysed using SPSS version 23.0 (SPSS, Chicago, IL). All data were weighted for statistical analyses to account for the complex multistage, stratified, and unequally weighted or clustered sampling design of the KNHANES VI. Appropriate sampling weighting factors were selected as specified from each national dataset. The *chi*-square test and independent *t* test were used to compare the characteristics of subjects in the periodontitis and non-periodontitis groups. Multivariate logistic regression analyses were applied to identify associations between regular walking and periodontitis after adjusting for potential confounders. Regression model 1 adjusted for age and gender. Individual income and level of education were added to regression model 2. Smoking and diabetes mellitus were added to regression model 3. Oral health behaviours were added to regression model 4. Other multivariate logistic regression analyses were performed to identify the association between periodontitis and socio-economic status after adjusting for potential confounders in the whole group, the non-regular walking group, and the regular walking group. In model 1, age and gender were adjusted for, and the effect of income on periodontitis was evaluated. The level of education was added to regression model 2. Smoking and diabetes mellitus were added to regression model 3. Oral health behaviours were added to regression model 4. *P* < 0.05 was considered to be statistically significant.

## Results

The characteristics of the subjects according to age and gender are shown in Table 1. The subjects who had periodontitis (mean: 54.3 years old) were significantly older than those who did not have periodontitis (mean: 42.4 years old). The proportion of males was significantly higher in the periodontitis group (58.0%) than in the non-periodontitis group (45.5%). The individual income and level of education were significantly different between the two groups. The subjects, who did not have periodontitis, were wealthier and more educated comparing to those who had periodontitis. The proportion of current or past smokers was significantly higher in the periodontitis group (54.0%) than in the non-periodontitis group (37.9%). With respect to oral-health behaviour, subjects who choose ‘yes’ for the use of dental floss and interdental brush were significantly lesser in the periodontitis group (interdental flossing: 13.5%, interdental brushing: 16.7%) than in the non-periodontitis group (interdental flossing: 27.8%, interdental brushing: 22.6%). Subjects who chose ‘yes’ for regular walking were also significantly lesser in the periodontitis group (35.8%) than the non-periodontitis group (43.3%).

**Table 1:** The characteristics of subjects in total group and by periodontitis.

	Total group		Periodontitis				*P*-value
	Unweighted N	Weighted % (95% CI)	No		Yes		
			Unweighted N	Weighted% (95% CI)	Unweighted N	Weighted % (95% CI)	
Age (years)	9728	45.9 (45.3–46.5)	6533	42.4 (41.7–43.0)	3195	54.3 (53.6–55.1)	<0.001^*^
Gender							
Male	4110	49.2 (48.1–50.2)	2466	45.5 (44.1–46.8)	1644	58.0 (56.3–59.7)	<0.001^†^
Female	5618	50.8 (49.8–51.9)	4067	54.5 (53.2–55.9)	1551	42.0 (40.3–43.7)	
Income							
Low	2318	24.7 (23.1–26.3)	1470	23.4 (21.7–25.2)	848	27.7 (25.5–30.1)	<0.001^†^
Middle low	2423	25.2 (23.8–26.7)	1572	24.5 (22.8–26.2)	851	27.0 (25.1–29.1)	
Middle high	2486	25.0 (23.6–26.5)	1735	25.8 (24.2–27.4)	751	23.2 (21.3–25.3)	
High	2446	25.1 (23.1–27.2)	1723	26.3 (24.1–28.7)	723	22.0 (19.7–24.6)	
Education							
≤Elemental school	1999	15.5 (14.3–16.8)	1069	11.5 (10.4–12.6)	930	25.2 (22.9–27.7)	<0.001^†^
Middle school	966	8.9 (8.2–9.7)	525	6.9 (6.2–7.6)	441	13.8 (12.2–15.5)	
High school	2993	38.0 (36.6–39.5)	2113	39.5 (37.8–41.2)	880	34.5 (32.2–36.8)	
≥University or college	2940	37.6 (35.8–39.4)	2289	42.2 (40.2–44.2)	651	26.5 (23.8–29.4)	
Smoking							
Never	5728	57.4 (56.3–58.6)	4164	62.1 (60.6–63.6)	1564	46.0 (44.1–48.0)	<0.001^†^
Current or past	3553	42.6 (41.4–43.7)	2085	37.9 (36.4–39.4)	1468	54.0 (52.0–55.9)	
Diabetes mellitus							
Normal	914	8.4 (7.8–9.2)	447	5.7 (5–6.4)	467	15.3 (13.8–16.9)	<0.001^†^
Impaired fasting glucose	1895	21.9 (20.7–23.1)	1119	18.8 (17.5–20.1)	776	29.6 (27.5–31.8)	
Diabetes	5447	69.7 (68.3–71)	4014	75.6 (74–77.1)	1433	55.1 (52.9–57.4)	
Interdental flossing							
No	7234	76.4 (75.1–77.6)	4593	72.2 (70.7–73.7)	2641	86.5 (84.7–88.0)	<0.001^†^
Yes	2047	23.6 (22.4–24.9)	1658	27.8 (26.3–29.3)	389	13.5 (12.0–15.3)	
Interdental brushing							
No	7504	79.1 (78.0–80.2)	4931	77.4 (76.0–78.7)	2753	83.3 (81.3–85.2)	<0.001^†^
Yes	1777	20.9 (19.8–22.0)	1320	22.6 (21.3–24.0)	457	16.7 (14.8–18.7)	
Regular walking							
Yes	3541	41.1 (39.6–42.5)	2477	43.3 (41.6–45.0)	1064	35.8 (33.5–38.1)	<0.001^†^
No	5355	58.9 (57.5–60.4)	3516	56.7 (55.0–58.4)	1839	64.2 (61.9–66.5)	

^*^Results were obtained by independent t-test. ^†^Results were obtained by *chi*-square test. CI means Confidence interval.

Table 2 shows the results of the logistic regression analyses to determine the presence of multivariable associations between periodontitis and regular walking after adjusting for age, gender, individual income, level of education, smoking, diabetes mellitus, and oral-health behaviour. The four logistic regression models were designed to adjust for covariates hierarchically. In all models, subjects who walked regularly showed significantly lower risks of periodontitis than subjects who did not. The adjusted odds ratio (OR) was 0.793 with 95% confidence interval (CI) of 0.699–0.898 for regular walking in model 4.

**Table 2:** Multivariable association between regular walking and periodontitis.

Regular walking	Model 1	Model 2	Model 3	Model 4
	N = 8,896	N = 8,846	N = 8,043	N = 8,042
Yes, OR (95% CI)	**0.762** **(0.678–0.857)**	**0.759** **(0.674–0.855)**	**0.787** **(0.695–0.892)**	**0.793** **(0.699–0.898)**
No	Reference	Reference	Reference	Reference

Response variable: Periodontitis.

Explanatory variable: Regular walking.

Model 1 was adjusted for age and gender.

Model 2 was adjusted for age, gender, individual income, and level of education.

Model 3 was adjusted for age, gender, individual income, level of education, smoking, and diabetes mellitus.

Model 4 was adjusted for age, gender, individual income, level of education, smoking, diabetes mellitus, dental flossing, and interdental brushing.

Bold denotes statistical significance at *P* < 0.05. OR means odds ratio. CI means confidence interval.

Table 3 shows the results of the logistic regression analyses for multivariable associations between periodontitis and socio-economic status in the non-regular walking and regular walking groups after adjusting for age, gender, level of education, smoking, diabetes mellitus, and oral-health behaviour. When both groups were analysed together, all models showed a significantly higher risk of periodontitis in the low (OR: 1.388, 95% CI: 1.215–1.711 in model 4) and middle low groups (OR: 1.314, 95% CI: 1.087–1.589 in model 4) comparing to high income group. Similar high risks of periodontitis with lower socio-economic statuses were also seen in all the models in the non-regular walking group and models 1, 3, and 4 in the regular walking group. A significant association between periodontitis and socio-economic status was not found in model 2 of the regular walking group.

**Table 3:** Multivariable association between individual income and periodontitis in the entire study group, non-regular walking, and regular walking groups.

OR (95% CI)	Total	Regular walking	
		No	Yes
**Model 1**			
Low	**1.518 (1.262–1.826)**	**1.566 (1.24–1.978)**	**1.389 (1.031–1.87)**
Middle low	**1.423 (1.2–1.687)**	**1.390 (1.122–1.723)**	**1.380 (1.049–1.816)**
Middle high	1.095 (0.925–1.296)	1.031 (0.839–1.267)	1.096 (0.839–1.43)
High	Reference	Reference	Reference
**Model 2**			
Low	**1.418 (1.169–1.72)**	**1.481 (1.165–1.883)**	1.301 (0.958–1.767)
Middle low	**1.341 (1.125–1.597)**	**1.332 (1.073–1.653)**	1.314 (0.991–1.742)
Middle high	1.039 (0.87–1.242)	1.001 (0.813–1.233)	1.065 (0.811–1.399)
High	Reference	Reference	Reference
**Model 3**			
Low	**1.414 (1.147–1.742)**	**1.423 (1.098–1.844)**	1.366 (0.995–1.874)
Middle low	**1.333 (1.102–1.612)**	**1.282 (1.013–1.621)**	**1.376 (1.029–1.841)**
Middle high	1.045 (0.863–1.266)	0.982 (0.783–1.231)	1.13 (0.85–1.501)
High	Reference	Reference	Reference
**Model 4**			
Low	**1.388 (1.125–1.711)**	**1.394 (1.074–1.810)**	1.345 (0.977–1.850)
Middle low	**1.314 (1.087–1.589)**	**1.267 (1.001–1.603)**	**1.362 (1.018–1.821)**
Middle high	1.037 (0.855–1.258)	0.981 (0.782–1.232)	1.114 (0.836–1.484)
High	Reference	Reference	Reference

Response variable: Periodontitis.

Explanatory variable: Individual Income.

Model 1 was adjusted for age and gender.

Model 2 was adjusted for age, gender, and level of education.

Model 3 was adjusted for age, gender, level of education, smoking, and diabetes mellitus.

Model 4 was adjusted for age, gender, level of education, smoking, diabetes mellitus, dental flossing, and interdental brushing.

Bold denotes statistical significance at *P* < 0.05. OR means odds ratio. CI means confidence interval.

## Discussion

This cross-sectional study assessed the effects of regular walking on periodontitis. Our study showed a significant association between regular walking and lower prevalence of periodontitis. Only one other study reported the relationship between walking and periodontitis. In this study done by Merchant *et al*.^28^, walking was assessed separately from other forms of physical activity, and walking was reported to be inversely associated with periodontitis after adjusting for age and smoking. However, this study only used data from people from the United States, and its subjects were health professionals. Using this, it is difficult to generalize the relationship between walking and periodontitis. We used the KNHANES VI complex sample data, and defined regular walking by the IPAQ. When age, gender, individual income, level of education, smoking, and diabetes mellitus were adjusted for (Model 4 of Table 2), the OR of developing periodontitis while regularly walking was 0.793 (95% CI: 0.699–0.898). This showed that regular walking had a preventive effect on periodontitis.

Bawadi *et al*.^21^ studied the relationship between periodontitis, physical activity, and healthy diet by randomly selecting 340 subjects and asking about their socio-demographic and clinical characteristics, anthropometric measurements, dietary assessment, and level of physical activity using the IPAQ. The subjects were divided into three categories: low, moderate, and high physical activity. The high physical active group had a significantly lower average plaque index, average gingival index, and average clinical attachment loss. They suggested that decreased physical activity and poor diet were significantly associated with periodontitis.

Al-Zahrani *et al*.^22^ also reported the relationship between physical activity and the prevalence of periodontitis. They used the NHANES III subjects (n = 2,521) and suggested that a high level of physical activity can prevent periodontitis. Anderson *et al*.^29^ evaluated the relationship between physical activity and periodontal pathogens. They reported that physical activity had a positive association with the antibodies in the orange and blue complex related to healthy periodontal states. Although they did not use regular walking as a separate effect modifier, these results are similar to the findings of our study and support the hypothesis that physical activity reduces the prevalence of periodontitis.

Three main mechanisms may explain the association between regular walking and periodontitis: oxidative stress, inflammation, and insulin resistance. Firstly, regular walking may decrease oxidative stress. obesity, diseases that are prone to people who do not even exercise lightly such as walking, decreased in infiltration of oxidized lipids into the lining of the blood vessel, which result in oxidative stresses in the blood vessel walls^30–32^. Secondly, regular walking could reduce vascular inflammatory markers^9,33^. Metabolic syndrome, prone to obesity, is the chronic inflammation caused by increased production of reactive oxygen species^34^. Regular walking is very effective in preventing metabolic syndrome^35^. Thirdly, regular walking have been reported to reduce HbA1c in diabetic patients^15^ and to significantly reduce the systolic blood pressure of subjects participating in the 6-month gait program^36^. Consequentially, these reduce the risk of developing periodontal diseases to have indirect common pathway that causes a reduction in inflammatory mediators^37^. As such, the effect of regular walking, in physical activity, was announced.

Based on these mechanisms, people with regular walking deficiency are prone to obesity and hypertension^16^, and these diseases are closely related to periodontal disease^30,38,39^. Although walking is not enough to reduce oxidative stress, inflammation, and insulin resistance immediately, the regular walking which we define was at least 1 time 30 minutes and more 5 days a week. This definition of regular walking was not easily achievable by ordinary people and this regular walking could be effective on prevention of obesity and hypertension^36,40^.

In our study, the results of logistic regression analyses showed that subjects who walked regularly had significantly lower risk (OR: 0.793, CI: 0.699–0.898) of periodontitis than those who did not. This result confirms that regular walking can have a positive effect on the health of the whole body, as well as oral health specifically.

We also found that regular walking may attenuate the relationship between periodontitis and low socioeconomic status. The non-regular walking groups were significantly associated with the low social and economic status seen in the below median income group. This was maintained even after all confounders had been adjusted for. No significant association was found between the social and economic status seen in the lowest income groups in the regular walking group after the confounders had been adjusted for in models 2, 3, and 4 of Table 3. These results can be interpreted as showing that regular walking alleviates the relationship between periodontitis and low socioeconomic status by reducing periodontitis since the ORs decreased in the regular walking group when compared with the non-regular walking group.

Generally, Periodontal disease is associated with health inequalities. People, who have high socio-economic statuses show a tendency to maintain good oral health whereas, those who have low socio-economic statuses show a susceptibility to periodontitis^41^. Economic inequality, in addition to predicting general morbidity and mortality, is also strongly related to unhealthy behaviours and habits^42^.

The socioeconomic status and other systemic factors, including physical activity, could be important factors associated with periodontitis^43^. However, oral health experts generally offer advice only on plaque control to people with periodontitis. Considering the results of this study, it may be clinically helpful to advice patients with periodontitis on the benefits of physical activity, especially regular walking.

There is no previous study that explores the association between the regular walking and periodontal heath inequalities. The results of this study suggest that promoting regular walking can promote oral health. However, further studies are needed because causal relationships between regular walking and periodontal health could not be discussed in this study. This would be useful for establishing a guideline for decreasing health inequalities with regard to periodontitis.

The following are the limitations of this study. Firstly, it had a cross-sectional design which does not allow determining the direction of the causal relationship between regular walking and periodontitis. Further studies that adopt a prospective design are needed for the same. Longitudinal studies should be done to verify the presence of a direct role of physical activity in preventing periodontitis and to determine its interactions with other factors that are known to affect periodontitis. Secondly, this survey was limited to Koreans and can hinder the generalization of the results. Thirdly, since the study was based on self-reported health status and physical activity, there might have been bias. Finally, since periodontitis was evaluated using the CPI, periodontitis could be over- or underestimated^44^. Generally, periodontal statuses are assessed by using clinical attachment level and pocket depth. However, CPI is an epidemiologic tool developed by WHO. The measurement of regular walking in the KNHANES data was based on IPAQ that has been widely used and has acceptable validity. Moreover, the data covers a large number of subjects, and the complex sampling design was considered during all analyses to overcome shortcomings.

Walking is a simple, safe, and cost-effective health behaviour that can reduce the prevalence of chronic diseases and reduce the cost of health care^45–47^. Our study supported the hypothesis that regular walking is associated to lower prevalence of periodontitis. We also found that regular walking can attenuate the relationship between periodontitis and low socioeconomic status.
